# Gentamicin-Releasing Mesoporous ZnO Structures

**DOI:** 10.3390/ma11020314

**Published:** 2018-02-22

**Authors:** Marco Laurenti, Valentina Cauda

**Affiliations:** Department of Applied Science and Technology, Politecnico di Torino, C.so Duca degli Abruzzi 24, 10129 Turin, Italy

**Keywords:** zinc oxide, mesoporous structure, gentamicin sulfate, drug delivery

## Abstract

Among metal oxides, zinc oxide (ZnO) is one of the most attractive materials thanks to its biocompatible and biodegradable properties along with the existence of various morphologies featuring piezoelectric, semiconducting and photocatalytic activities. All of these structures were successfully prepared and tested for numerous applications, including optoelectronics, sensors and biomedical ones. In the last case, biocompatible ZnO nanomaterials positively influenced cells growth and tissue regeneration as well, promoting wound healing and new bone formation. Despite showing high surface areas, ZnO morphologies generally lack an intrinsic mesoporous structure, strongly limiting the investigation of the corresponding drug loading and release properties. Within this scope, this study focuses on the adsorption and release properties of high surface area, mesoporous ZnO structures using gentamicin sulfate (GS), a well known antibiotic against bacterial infections especially in orthopedics. The particular ZnO morphology was achieved starting from sputtered porous zinc layers, finally converted into ZnO by thermal oxidation. By taking advantage of this mesoporous framework, GS was successfully adsorbed within the ZnO matrix and the kinetic release profile evaluated for up to seven days. The adsorption of GS was successfully demonstrated, with a maximum amount of 263 mg effectively loaded per gram of active material. Then, fast kinetic release was obtained in vitro by simple diffusion mechanism, thus opening further possibilities of smart pore and surface engineering to improve the controlled delivery.

## 1. Introduction

Zinc oxide (ZnO) is a wide band-gap metal oxide semiconductor (3.37 eV) characterized by the presence of piezoelectric [1] and pyroelectric [2] properties, biocompatibility [3,4] and photocatalyitic activity [5,6]. This metal oxide may be prepared in a huge amount of morphologies and shapes, like thin films [7,8], nanowires [9], nanorods [10], nanobelts [11], nanoparticles [12] and flower-like structures [13]. All of the mentioned ZnO structures are easily obtained by following wet and dry synthesis approaches, such as sol-gel and hydrothermal routes as well as vapor-phase deposition methods.

Because of its multifunctional properties and the existence of various micro- and nanostructured morphologies, ZnO has gained considerable attention and for different application fields. The photocatalytic activity of ZnO was successfully demonstrated in numerous cases and for different morphologies [5,6,14,15,16,17], finding application for water splitting [18,19] and degradation of dye pollutants for waste-water treatments [20,21]. ZnO nanostructures with promising piezoelectric and semiconducting properties were successfully obtained as well, resulting in novel smart materials applicable for new generation energy harvesting systems [22,23,24,25,26] and sensing applications [8,23,27,28,29,30,31]. Concerning the biomedical field, ZnO was extensively investigated as active material for biosensing applications [32,33,34]. To this purpose, many works highlighted the promising use of ZnO nanostructures for the detection of different biomoieties, like glucose [35,36,37], acid uric [38,39,40] and proteins [31,41]. More recently, the interest in studying ZnO nanomaterials for tissue engineering came up as well [4]. In particular, the ability of ZnO nanosheets, nanoflowers and nanorods to actively promote the growth and proliferation of several cells line was reported [42,43,44], together with the successful demonstration of ZnO proangiogenic [45] and osteogenic activities [43], particularly desirable for wound healing and the formation of new bone tissue.

Despite showing high surface areas, many ZnO morphologies suffer from a major drawback, the lack of an intrinsic mesoporous structure. Especially for drug delivery applications, this aspect strongly limited their investigation as possible alternatives to other existing materials, like mesoporous silica nanoparticles (MSNs) [46,47]. However, ZnO on its own is very sensitive towards pH variations. This property was successfully exploited in the case of ZnO quantum dots [48], which were also combined to doxorubicin-loaded MSNs and used as nanolids to cover silica nanopores [49]. In this way, under specific pH variations ZnO nanolids allowed the opening of the pores and the controlled delivery of doxorubicin. Exploiting ZnO decomposition in acidic environments, ZnO nanoparticles were also combined into biodegradable polymers, resulting in core-shell structures with high loading of doxorubicin amounts and pH-triggered release properties [50]. Mesoporous ZnO nanospheres showing biocompatible properties were also successfully synthesized by a soluble-starch-insertion method. The mesoporous structure effectively allowed for captopril drug molecules to be efficiently loaded and released during in vitro experiments [51]. Alternatively, a porous hexagonal ZnO nanodisc structure prepared by microwave synthesis was proposed as a nanocarrier for drug delivery applications. After the chemical modification of the outer ZnO surface to improve the chemical stability and making loading more efficient, the proposed system showed promising results for the targeted, pH-triggered delivery of the anticancer drug against breast cancer cells [52]. Dandelion-like mesoporous ZnO capsules mainly formed by ZnO nanorods or nanoparticles were proposed as well [53]. Also in this case, interesting drug delivery properties were obtained and expressed in terms of doxorubicin loading and release.

In this work, mesoporous ZnO structures were considered as active material and the related drug loading and release properties evaluated. Gentamicin sulfate (GS), a bactericidal aminoglycoside antibiotic, was selected as a drug molecule because of its wide use in the treatment of bacterial infections like osteomyelitis. The existence of a mesoporous framework with specific physical and chemical properties allowed the successful adsorption of GS within the ZnO matrix. The kinetic release profile was then evaluated in vitro for up to seven days. The results showed that promising GS adsorption could be obtained within 2 h of uptake experiment, with a maximum amount of 263 mg effectively adsorbed per gram of active material. Then, by simple diffusion mechanism, fast GS release was observed during in vitro experiments, as 90% GS delivery occurred within the first 2 h. These first results point out the well-known need in mesoporous systems to further engineer the pore chemical structure or entrance [47,54,55] in order to achieve a smart and even remote control on the drug delivery kinetics.

## 2. Results

**Figure 1:**
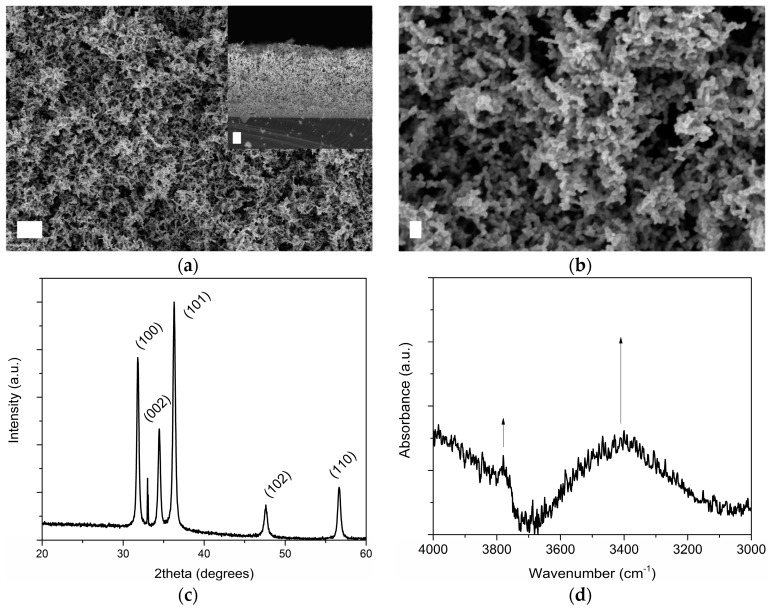
Surface morphology of mesoporous zinc oxide (ZnO) structures obtained by Field-Emission Scanning Electron Microscope (FESEM): (**a**) top and cross-section views—scale bar is 1 μm; (**b**) FESEM image at higher magnification—scale bar is 100 nm; (**c**) X-ray diffraction pattern; (**d**) infrared spectrum.

The surface morphology and cross-sectional structure of mesoporous ZnO structures grown on silicon (Si) substrates are shown in Figure 1 and were investigated by means of Field Emission Scanning Electron Microscopy (FESEM). The presence of a porous nanobranched network made of elongated, nanocrystalline ZnO grains interconnected to each other was noticed, together with the formation of cavities (see Figure 1b). The average thickness of the ZnO layer was around 9 μm. The degree of porosity was previously estimated [56] and confirmed the existence of a mesoporous framework with a discrete specific surface area (14 m^2^·g^−1^, pore volume 0.095 cm^3^·g^−1^), while an average pore diameter of 27 nm could be estimated according to Wheeler equation [57]. Figure 1c,d show the X-ray Diffraction (XRD) pattern and infrared (IR) spectrum of the mesoporous ZnO matrix. The sharp and intense XRD peaks positioned at 31.80°, 34.45° and 36.29° 2θ angles belong to (100), (002) and (101) crystal planes of hexagonal wurtzite ZnO, respectively (Joint Committee on Powder Diffraction Standards-International Centre for Diffraction Data (JCPDS-ICDD) database, card no. 89–1397). Other minor contributions at 47.61° and 56.67° were also detected and due to reflections coming from (102) and (110) wurtzite crystal planes. On the other side, IR spectroscopy highlighted the presence of a hydroxyl-rich surface, witnessed by the absorbance band at 3750 cm^−1^ due to stretching mode of free O−H, as well as by the broad band lying in the range 3600–3100 cm^−1^ and due to OH of water of crystallization.

**Figure 2:**
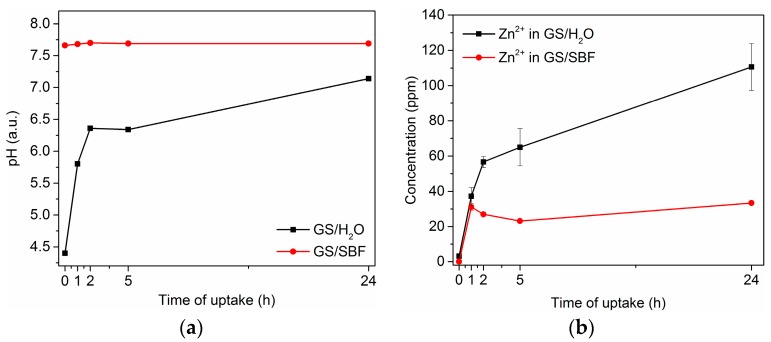
Comparison of gentamicin sulfate (GS) loading experiments performed by using different uptake solutions, i.e., GS/H_2_O vs. GS/SBF: (**a**) time evolution of pH; (**b**) ICP-MS release profile for Zn^2+^ ions. Error bars for GS/SBF fall within the resolution limit of instrument.

The gentamicin sulfate (GS) adsorption properties of mesoporous ZnO structures were investigated by soaking the samples in two separate uptake solutions and for different times (1, 2, 5 and 24 h): GS in water (GS/H_2_O) and GS in Simulated Body Fluid (GS/SBF). SBF is a buffered solution simulating the inorganic composition of human plasma and derives from the formulation of Kokubo [58]. Figure 2 summarizes the effect of using GS/H_2_O and GS/SBF in the drug loading, remarking a degradation behavior of the ZnO matrix during GS loading in water solution. The time evolution of the corresponding pH value is shown in Figure 2a, while Figure 2b reports the release profile for Zn^2+^ ions against the soaking time, obtained by Inductively Coupled Plasma-Mass Spectrometry (ICP-MS). Actually, in the case of GS/H_2_O, the starting acidity of the loading solution (initial pH 4.4) led to a remarkable degradation of the mesoporous structure, as witnessed by the increasing amount of Zn^2+^ ions coming from the ZnO matrix and released in the uptake solution. The highest concentration value for Zn^2+^ (~110 ppm) was observed for the longest soaking time (24 h). The release of Zn^2+^ ions also induced a continuous increase of the pH value during time, as visible in Figure 2a. On the contrary, no remarkable degradation of the ZnO matrix occurred during drug loading by using GS/SBF buffered solution (initial pH 7.4), except for the beginning of the loading experiment; within the first hour, release of Zn^2+^ ions were slightly comparable to the GS/H_2_O case, but then it stabilized to a nearly constant level (~25–35 ppm) for the remaining part of the loading experiment. This time evolution profile of Zn^2+^ agreed with the corresponding evolution of pH; actually, the pH in the SBF buffered solution did not change up to 24 h.

**Figure 3:**
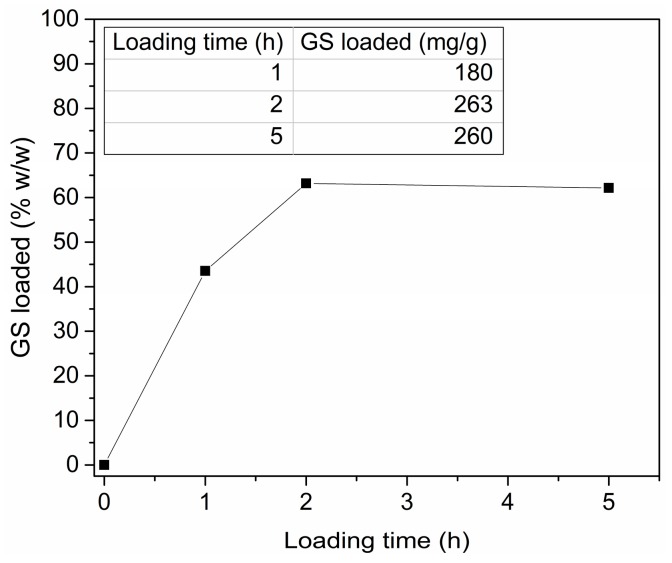
Gentamicin sulfate loading against time.

By considering the strong ZnO degradation observed for the GS/H_2_O solution, the buffered GS/SBF one was selected as the optimal choice for efficient GS loading, as it prevented the dissolution of the mesoporous framework. Figure 3 shows the amount of GS effectively adsorbed within the ZnO matrix against the loading time. By changing the soaking time from 0 to 2 h, the amount of GS loaded within mesoporous ZnO increased accordingly, reaching a maximum value of 263 mg adsorbed per gram of active material. If the loading time was further increased up to 5 h, no additional drug adsorption was observed, as well as even up to 24 h (data not reported). Therefore, 2 h was selected as the optimal soaking time to load the maximum amount of drug. 

**Figure 4:**
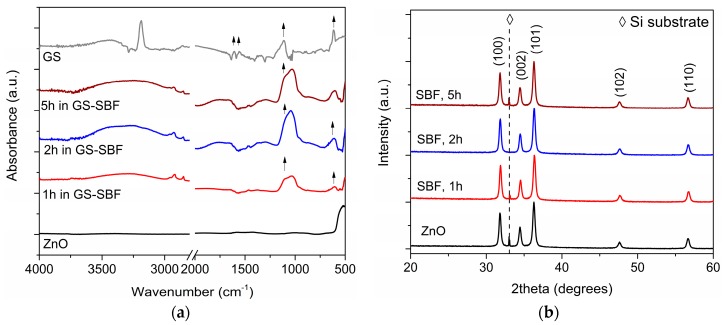
Structural and compositional characterization of mesoporous zinc oxide structures after loading gentamicin sulfate in SBF: (**a**) infrared spectra; (**b**) X-ray diffraction patterns.

The effective GS adsorption was also verified by IR spectroscopy. Figure 4a shows the IR spectra collected for ZnO samples after loading GS in SBF for up to 5 h. A broad absorption band in the range 3500–3100 cm^−1^ was observed in all the cases and due to –OH vibrational modes. The presence of GS was represented by the appearance of the sulfate band at 609 cm^−1^ and of C–O–C stretching mode at 1080 cm^−1^. However, this last one was very close and partially overlapped with the intense PO_4_^3−^ band lying in the range 1100–1000 cm^−1^ and due to the interaction of mesoporous ZnO with SBF solution. Figure 4b shows the corresponding XRD patterns. No additional contributions were detected while strong diffraction peaks belonging to hexagonal wurtzite ZnO were still present, independently of the soaking time. This confirmed that GS loading in SBF did not alter the structural properties of the mesoporous ZnO framework, as the starting crystalline structure was completely preserved.

**Figure 5:**
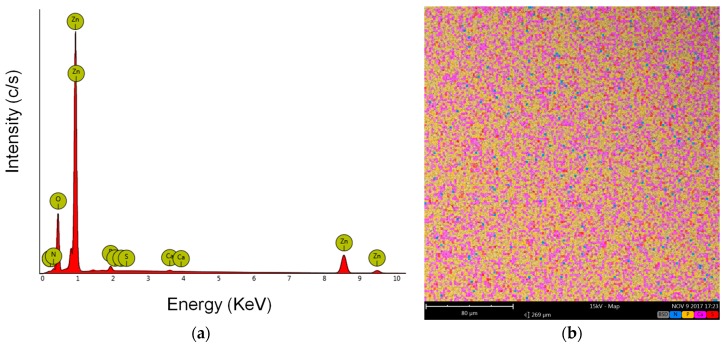
Energy Dispersive Spectroscopy (EDS) analysis on mesoporous ZnO matrix after loading gentamicin sulfate in SBF for 2 h: (**a**) EDS spectrum; (**b**) EDS map for calcium, phosphorous, sulfur and nitrogen elements.

After soaking ZnO sample in GS/SBF solution for 2 h, i.e., the optimal loading time, the effective GS adsorption was qualitatively confirmed also by Energy Dispersive X-ray Spectroscopy (EDS) analysis. Apart from Zn (45.75 at %) and O (48 at %), the presence of S (0.03 at %), N (4.12 at %), P (1.73 at %) and Ca (0.37 at %) elements was noticed, as shown in EDS spectrum of Figure 5a. The map corresponding to the distribution of the detected elements is represented in Figure 5b. Zn and O were the most abundant ones and uniformly distributed on the whole sample area. Therefore, these were not included in the map. The presence of S (red) and N (blue) was then considered a qualitative indication of GS adsorption, while P (pink) and Ca (yellow) were detected due to Calcium Phosphates compounds’ precipitation deriving from the interaction of ZnO with SBF solution, as evidenced by the IR spectra in Figure 4a.

**Figure 6:**
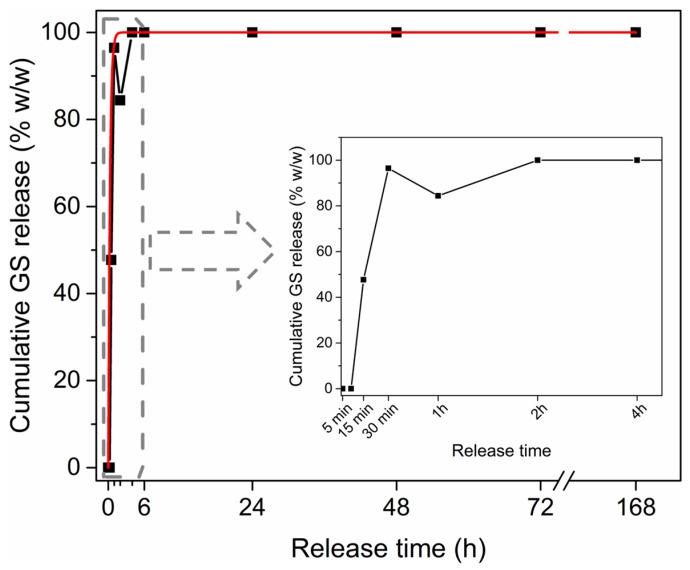
Kinetic release profile for gentamicin sulfate in SBF up to 7 days. Inset shows the release profile within the first 4 h.

**Figure 7:**
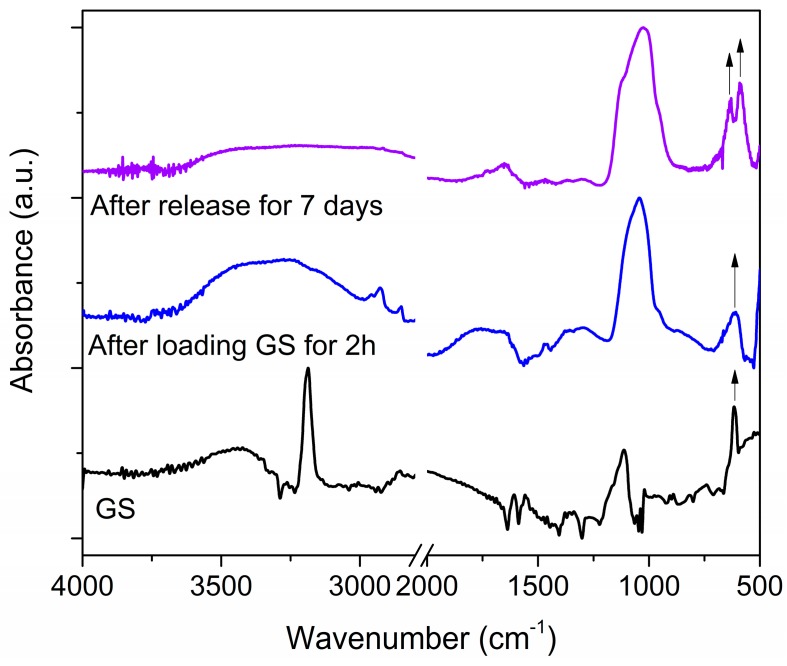
Infrared spectra obtained for gentamicin sulfate (GS), mesoporous ZnO structures after GS loading for 2 h, mesoporous ZnO after GS release in SBF performed for up to seven days.

After loading in SBF for 2 h, the GS kinetic release profile was monitored against time, for up to seven days in SBF at 37 °C. Figure 6 shows an important burst delivery, with around 90% GS released within the first 2 h, then approaching 100% after 24 h and being stable around this value for up to seven days. The release profile followed a zero-order exponential decay law (see the red fitting curve), with a release rate constant of 3.69 ± 2.25 s^−1^, representing that GS release out from the mesoporous matrix was driven by simple diffusion mechanism. An IR spectrum was also acquired on the GS-loaded mesoporous ZnO at the end of the release experiment, i.e., after seven days, as shown in Figure 7. The absence of the sulfate band at 609 cm^−1^ confirmed the complete GS release. At the same time, the rise of PO_4_^3−^ vibrational modes in the range 600–550 cm^−1^ were due to CaP formation after the prolonged exposure of the sample to SBF, as previously observed from EDS analyses.

## 3. Discussion

Within the wide plethora of nanomaterials investigated for drug-delivery applications, the interest in considering ZnO structures was somehow limited. This was mainly due to the lack of an intrinsic mesoporous structure, which could prevent drug molecule adsorption and release. Low-dimensional ZnO structures, i.e., quantum dots and spherical nanoparticles, were successfully loaded alone or in combination with MSNs, with anticancer drugs like doxorubicin [49,50,52,53]. The general mechanism allowing for the controlled release of drug was driven by the sensitivity of ZnO to pH variations [59]. In the present study, an alternative way to prepare mesoporous ZnO structures showing drug adsorption and release properties was successfully demonstrated as well. The mesoporous ZnO matrix was prepared by a two-step synthetic approach, involving the sputtering deposition of porous metallic zinc layers and their following thermal oxidation. Gentamicin sulfate was effectively loaded within the ZnO matrix, thanks to the high-surface area of the mesoporous framework combined with the presence of hydroxyl groups. Both of these aspects allowed the drug molecule to be efficiently loaded. An important drawback for ZnO nanomaterials is the low chemical stability when surrounded by biological fluids [12]. This aspect was confirmed also in the present case. Depending on the GS loading solution, the mesoporous ZnO matrix showed different behaviors and hence drug loading efficiencies. When GS/H_2_O acidic solution was considered, drug loading was strongly limited by degradation phenomena affecting the mesoporous ZnO matrix. This was represented by the Zn^2+^ release profile, showing that strong degradation of the porous structure occurred during uptake experiments in GS/H_2_O. Therefore, drug loading was also performed considering a buffered GS/SBF solution. In this case, no remarkable changes in Zn^2+^ concentration were observed after 24 h. Hence, using a buffered GS/SBF solution allowed for strongly limiting the undesirable degradation of the mesoporous ZnO matrix, maximizing GS loading at the same time.

All of the results showed that high surface area, crystalline and mesoporous ZnO structures could effectively serve as a mesoporous matrix for GS adsorption. By considering degradation phenomena generally affecting ZnO, suitable uptake conditions were selected, allowing the loading of 263 mg GS per gram of active material. Finally, the GS-loaded mesoporous ZnO structures showed a fast kinetic delivery, with around 100% drug being released within a few hours by a simple and obvious diffusion mechanism. 

By considering these promising findings, new future applications of mesoporous ZnO structures as short-term type therapeutic devices could be envisioned. For example, their use as antibiotic-releasing coatings might be exploited to design drug-eluting cardiovascular or ureteral stents, bone and periodontal implants. Indeed, bacterial infections affecting the insertion of implants still represent one of the main problems and should be treated in an efficient and short time manner.

In the case of more time-controlled release, we envision the possibility of further engineering the chemical and physical structure of the ZnO mesopores or their entrance with smart coatings, biodegradable polymer layers or stimuli-triggered compounds. These strategies will prevent an uncontrolled and burst release of the drug from the whole mesoporous system, delaying the drug rate to several hours and even days. In contrast, the delivery of the therapeutic agent can be activated in a precise and time-controlled manner upon a specific stimulus. Ongoing studies are being carried out to investigate the antibacterial efficacy of the proposed mesoporous ZnO structures in vitro. Moreover, the citocompatibility of this novel mesoporous ZnO nanostructure towards living healthy cells from epithelial and bone tissues is still under investigation.

## 4. Materials and Methods 

*Synthesis of mesoporous zinc oxide (ZnO) structures.* Mesoporous ZnO samples (3 mg each) were prepared by following a two-step synthetic approach, as previously described [7]. In the first step, a metallic Zn layer was deposited at room temperature on silicon (Si) substrates (~1 cm^2^ area) by radio-frequency magnetron sputtering for an overall deposition time of 4 h. Then, thermal oxidation of the Zn-coated Si samples was performed in a muffle furnace at 380 °C (ramp rate 150 °C/h) in air for 2 h. Before Zn deposition, the Si substrates were cleaned in an ultrasonic bath of acetone and ethanol (10 min for each washing cycle) and dried under nitrogen flow.

*Gentamicin sulfate (GS) adsorption and release experiments.* GS powders were dissolved either in bidistilled water or simulated body fluid (SBF) at room temperature, under continuous stirring conditions (360 rpm) for 30 min. SBF salt solution was prepared according to Kokubo’s protocol [58]. Both GS/H_2_O and GS/SBF uptake solutions had a final concentration of 250 μg/mL. Adsorption of GS was performed at room temperature by soaking the mesoporous ZnO/Si samples for different times (1 h, 2 h, 5 h and 24 h) in a plastic tube filled with the uptake solution (5 mL), under orbital shaking conditions (160 rpm). After GS uptake, all the samples were washed with bidistilled water and air-dried overnight. 

GS release experiments were carried out by soaking the GS-ZnO/Si samples in a plastic tube filled with SBF (10 mL), in orbital conditions (160 rpm) at 37 °C for up to seven days. At specific points of time (5 min, 15 min, 30 min, 1 h, 2 h, 4 h, 6 h, 24 h, 48 h, 72 h, 7 days), 350 μL aliquot was collected from the release solution, centrifuged at 20,000× *g* for 5 min and analyzed by UV-Vis spectroscopy. The drug release profile was then obtained by considering the characteristic GS absorbance peak at 251 nm. This was compared with a calibration curve obtained from the UV absorbance values at 251 nm of a series of GS dilutions in SBF (from 5 to 1000 μg/mL). The amount of drug at each release time *w_t_* was obtained according to the following equation:

*w_t_* = *C_t_* × *V_t_*^−1^

where *C_t_* is the concentration of the solution collected at each release time *t_i_* (being *i* an integer *=* 1, 2, …, *n*), while *V_t_* is the residual volume of the release solution at that time, i.e., the starting release volume (10 mL) depleted at each time point *t_i_* of a fixed volume (*ΔV_i_* = 350 μL). The cumulative GS release profile was then obtained according to this equation:

*w_t_* (%) = (*w_t_* × *w*_0_^−1^) × 100

where *w*_0_ is the starting amount of GS loaded on the mesoporous matrix. 

*Sample characterization.* The morphology of the samples was evaluated by means of Field-Emission Scanning Electron Microscope (FESEM, Supra^®^40, Carl Zeiss AG, Oberkochen, Germany). X-ray Diffraction measurements were performed by a Panalytical X’Pert PRO diffractometer in Bragg-Brentano configuration, equipped with a Cu Kα monochromatic radiation (λ = 1.54059 Å) as X-ray source. The chemical composition was investigated by Energy Dispersive Spectroscopy (EDS), using a desktop SEM Phenom XL (Phenom-World B.V. part of Thermo Fisher Scientific, Eindhoven, The Netherlands) equipped with an EDS analyzer. UV-Vis absorbance spectra were collected in the range 200–285 nm, by means of a double-beam Varian Cary 5000 UV-vis-NIR spectrophotometer (Milan, Italy). UV analysis of drug solutions were carried out in a quartz cuvette, with an optical path length of 1 mm, analyzing a volume of 350 μL. All of the UV spectra were background subtracted. IR spectroscopy was carried out with a Nicolet 5700 FTIR Spectrometer (ThermoFisher, Waltham, MA, USA), equipped with a room temperature working L-alanine doped triglycine sulfate (DLaTGS) detector. All of the spectra were background subtracted and acquired with 2 cm^−1^ resolution and 64 scans accumulation. The release profile for Zn^2+^ element was determined with an Inductively Coupled Plasma Mass Spectrometer analyzer (ICP-MS, mod. 7500cc, Agilent Technologies, Milan, Italy).
